# Pathophysiological Mechanisms in Sclerosing Skin Diseases

**DOI:** 10.3389/fmed.2017.00120

**Published:** 2017-08-18

**Authors:** Beate Eckes, Fang Wang, Pia Moinzadeh, Nicolas Hunzelmann, Thomas Krieg

**Affiliations:** ^1^Department of Dermatology, University of Cologne, Cologne, Germany; ^2^Center for Molecular Medicine (CMMC), Cologne, Germany; ^3^Cluster of Excellence in Cellular Stress Responses in Aging-Associated Diseases (CECAD), Cologne, Germany

**Keywords:** systemic sclerosis, scleromyxedema, scleredema, stiff skin syndrome, nephrogenic systemic fibrosis, extracellular matrix, growth factor activation

## Abstract

Sclerosing skin diseases represent a large number of distinct disease entities, which include systemic sclerosis, localized scleroderma, and scleredema adultorum. These pathologies have a common clinical appearance and share histological features. However, the specific interplay between cytokines and growth factors, which activate different mesenchymal cell populations and production of different extracellular matrix components, determines the biomechanical properties of the skin and the clinical features of each disease. A better understanding of the mechanisms underlying these events is prerequisite for developing novel targeted therapeutic approaches.

## Introduction

Sclerosing skin diseases are a very heterogeneous group of diseases, which are characterized by excessive accumulation of extracellular matrix (ECM) constituents in the dermis or the subcutaneous tissue (Table 1). The common clinical characteristic is skin hardening often associated with thickening of the skin and firm adherence to the underlying fascia.

**Table 1:** Heterogeneity of sclerosing skin diseases.

Groups	Subsets	Variants
Systemic sclerosis	Diffuse cutaneous systemic sclerosis Limited cutaneous systemic sclerosis SSc overlap SSc sine scleroderma	
Localized scleroderma and its subsets	Limited form	Plaque type Guttata morphea Atrophoderma of Pasini and Pierini
	Generalized form	Generalized localized scleroderma Disabling pansclerotic morphea
	Linear form	Linear localized scleroderma En coup de sabre Parry Romberg syndrome
	Deep form Mixed form Eosinophilic fasciitis	
Scleredema adultorum	Type I postinfectious Type II with paraproteinemia Type III with diabetes mellitus	
Other	Scleromyxedema Nephrogenic systemic fibrosis Sclerodermiform porphyria cutanea tarda Stiff skin syndrome Toxic oil syndrome and others	

This group of diseases includes systemic sclerosis with its main subsets, the diffuse and the limited form (1–7). Overlap syndromes are complex diseases, also characterized by sclerosis of the skin as in systemic sclerosis but also associated with additional symptoms of other autoimmune diseases such as lupus erythematosus, dermatomyositis, Sjörgen’s syndrome, or rheumatoid arthritis (8). Localized scleroderma remains limited to the skin and also occurs in different subsets covering a wide spectrum of clinical aspects (2, 9). Some patients have minor involvement of the skin with a few fibrotic areas, whereas others show extensive involvement of large areas of the integument, which can lead to severe contractures and disabilities. The clinical diagnosis is easy if all characteristic symptoms are present. However, sclerodermatous features also occur in patients with scleromyxedema, scleredema, and sometimes in paraneoplastic processes. Also patients with porphyria cutanea tarda can develop an extensive sclerosing skin involvement. Therefore, all these diagnoses have to be considered in the differential diagnosis as well as other metabolic diseases such as amyloidosis or scleredema diabeticorum including Stiff skin syndrome, which is a rare inborn disease that develops extensive thickening of the dermis due to an underlying mutation in fibrillin, a major component of the elastic fiber network in the dermis (10–13). The clinical characteristics of all these diseases are similar to severe localized scleroderma but distinct, and the disease entities can be clearly distinguished. There are also some common but distinct features at the histological and ultrastructural level.

## Pathophysiology of Scleroderma and Related Diseases

**Figure 1:**
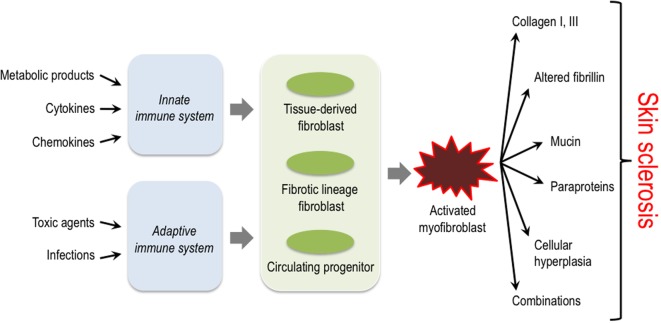
Triggers, key cellular players and structural proteins involved in skin sclerosis. Activation of the innate as well as the adaptive immune system by diverse triggers is thought to lead to activation of different fibroblast progenitors and lineages. These then convert to the myofibroblast that plays a central role in producing excessive amounts of extracellular matrix and other protein components typically associated with certain subsets of sclerosing skin diseases.

The activation of mesenchymal cells in the dermis is a common pathophysiological hallmark in all these diseases; however, the initial trigger mechanisms, the origin of these cells, and also the exact characteristics of the biosynthetic products (proteins, cytokines, proteases, etc.) that are deposited into the dermis by these activated mesenchymal cells, differ considerably depending on the specific disease entity (Figure 1) (14–17).

In this mini-review, we want to discuss the mechanisms leading to the excessive deposition of various ECM constituents in these different diseases and highlight both the common but also the disease-specific characteristics.

The histology of skin lesions in patients with localized scleroderma, systemic sclerosis, or the overlap syndromes reveals the presence of early perivascular lymphohistiocytic infiltrates that are probably subsequent to an early endothelial damage (1, 5, 18). These consist of activated mononuclear cells that release a large number of proinflammatory and fibrogenic cytokines. In some of these diseases, e.g., the Shulman syndrome, eosinophils represent a high percentage of cells in the early inflammatory infiltrates. Also, macrophages are thought to play an important role in the initial phases of these diseases. These observations are supported by recent gene profiling results from the analysis of skin biopsies (19, 20).

Following the initial inflammatory reaction, fibrogenic cytokines are generated and released from the cells. These include transforming growth factor beta (TGFβ), connective tissue growth factor, platelet-derived growth factor, the interleukins, and others, which are thought to be the key players contributing to the activation of fibroblasts and/or mesenchymal cells that then produce excessive amounts of ECM molecules (5, 18). Although it is still unclear whether tissue-resident fibroblasts, bone marrow-derived circulating progenitor cells, or tissue-derived progenitor cells are activated, the resulting cell type is called “myofibroblast,” and it is characterized by the incorporation of alpha smooth muscle actin into prominent stress fibers (21, 22). These cells are capable of producing excessive amounts of ECM and exerting considerable force against this matrix. They were first identified at the ultrastructural level by their extraordinary cytoskeletal properties in healing wounds, where they are responsible for reconstituting the dermal tissue (23).

## Role of the ECM and Biomechanics in the Microenvironment

Alterations in quantity as well as organization of the ECM are a prime hallmark of sclerosing skin disorders. In physiological conditions, the ECM is a highly organized structure containing a large number of different constituents. These include fibrils such as fibrillins and many different types of collagens mixed with non-fibrillar components such as proteoglycans, fibulins, fibronectin, matrilins, thrombospondins, and many others (24–27). The relative amounts of these components and the interactions among the diverse constituents determine the type of extracellular networks (28), which in turn control the biomechanical quality, the rigidity, and stiffness of the connective tissues (29, 30).

The interstitial fibrillar collagens represent the main structural component of connective tissues; their macromolecular organization, however, is regulated by their interaction with the fibril-associated collagens and with non-collagenous ECM components (31, 32). Biosynthesis of collagens is complex; the triple helical molecules are produced in a precursor form that contains the characteristic triple helix featuring repeats of the amino acids glycine-x-y, where y, is either proline or hydroxyproline. Intracellularly, several posttranslational modifications take place, which include the hydroxylation of prolyl and lysyl residues (33). The molecular mechanisms how these large collagen molecules are secreted are not fully understood and may comprise specialized carriers (34, 35). Once in the extracellular space, procollagen propeptides present at both ends of the molecules are removed to enable the aggregation of individual molecules into higher order supramolecular structures. Collagen fibrils are then cross-linked to form insoluble structures that provide tensile strength and stability to tissues (36). This requires the activity of the intracellular lysyl hydroxylase and the lysyl oxidase in the extracellular space, which is responsible for cross-link formation between individual collagen molecules (37, 38). The activity of these posttranslationally modifying enzymes depends on the specific cellular conditions and can be modulated by inflammatory cells and certain cytokines (39). The amount and the type of cross-link vary considerably in soft and hard tissues and are among the major determinants of tissue stiffness. Hence, soft connective tissues such as skin are characterized by the hydroxylysinorleucine cross-link type, whereas the dihydroxy lysine-norleucine (DHLNL) type predominates in bone and cartilage (31). Interestingly, the DHLNL type of cross-link is also a prominent feature of the collagen network in hardened fibrotically altered skin (40–42). More recently, it also became evident that tissue stiffness and the transduction of mechanical force regulate cellular activity including cell motility (43) and myofibroblast formation (44). Thereby, enhanced tissue stiffness contributes to the sustainability of a fibrotic process.

## The Stiff Skin Syndrome as a Model

Interestingly, fibrotic reactions and enhanced tissue stiffness can arise independently of the early endothelial cell damage and the resulting perivascular lymphohistiocytic infiltrate or other inflammatory conditions. This is well illustrated by the stiff skin syndrome, a rare inborn disease characterized by excessive accumulation of ECM in the fascia and the skin. Dietz and co-workers showed that this syndrome is caused by mutations in the 8-cysteine domain of fibrillin-1 (10). Fibrillin monomers are large glycoproteins of 350 kilodaltons, which assemble into microfibrils with small diameter and are important constituents of elastic fibers (12). In stiff skin syndrome, however, the altered fibrillin microfibrils form large aggregates, in which the microfibrils display an abnormal ultrastructure. In addition, the mutation in the 8-cysteine domain affects the integrin-binding site in fibrillin and leads to an aberrant binding of cells to the fibrillin microfibrils. Actually, introducing this mutation into mice is sufficient to trigger a stiff skin syndrome like phenotype in these mice (11).

An independent clue that modified tensile strength due to mutations in fibrillin can lead to fibrotic reactions came from the identification of the molecular defect in the Tight skin mouse, a model that is characterized by prominent skin fibrosis and that has been extensively investigated to better understand scleroderma (45). Here, a large duplication in the fibrillin-1 gene also leads to microfibrils with an altered ultrastructure of the connective tissue in the dermis (46). However, how the altered fibrillin molecules lead to the clinical phenotype has remained unclear. It has been postulated that the duplication might enhance the binding capacity of the altered fibrillin microfibrillar network for cytokines, especially for TGFβ, which could result in higher local TGF-β concentrations and increased local activity following its release from the ECM. This has supported the concept that ECM-bound growth factors and cytokines regulate local inflammatory reactions and that the ECM can provide storage places for such factors. Binding to ECM can both activate proforms of growth factors and inactivate biologically active mediators (47). This seems to represent a general concept explaining how key cytokines and growth factors can rapidly be made available upon demand such as after tissue injury. This concept is illustrated by several examples including fibroblast growth factor (48, 49) and the family of bone morphogenetic proteins (50–52). It is probably best documented for TGFβ that can be released in an activated form from fibrillin microfibrils and other ECM structures by mechanical tension (53–55).

## Nephrogenic Systemic Fibrosis

Nephrogenic systemic fibrosis is a very rare sclerosing disease that is frequently seen in patients with impaired renal function (56). Usually symmetrical sclerotic plaques develop, often at the lower legs and stretching over the joints, which can result in severe contractures. Gadolinium has been identified as the main external trigger of this disease (57). However, the exact pathophysiological mechanism is still unclear and a matter of debate. There is the characteristic appearance of CD34-positive spindle cells as well as specifically activated macrophages. This has led to the hypothesis that gadolinium triggers the activation of dendritic cells and/or macrophages to release cytokines that then induce the accumulation and activation of fibroblasts and progenitor cells (56, 58). Both, the clinical appearance as well as the characteristic histology enable a clear distinction from localized or systemic scleroderma and represent other pathophysiological events leading to the clinical symptom of sclerodermatous skin.

## Scleredema and Scleromyxedema

Obviously, in patients with scleredema and scleromyxedema, the histological alterations and the clinical appearance of the skin are quite distinct. Scleredema, which occurs in three types (postinfectious, associated with paraproteinemia or associated with diabetes mellitus), is characterized by excessive deposition of mucin in the dermis (59). There is also fragmentation of the elastic fibers, but no evidence of endothelial damage with the resulting perivascular infiltrates such as those seen in scleroderma. The biomechanical appearance of the skin in the involved areas especially on the back of the patients is highly characteristic and allows a rapid diagnosis.

Dermal mucin deposition is also a hallmark of scleromyxedema, which is associated with the presence of monoclonal paraproteins and severe extracutaneous involvement (60, 61). These patients develop multiple waxy papules and fibrotic plaques. The pathogenesis is not yet understood, and although monoclonal gammopathy is often associated with this disease, its pathogenetic role is unclear. Histologically, proliferating spindle-like fibroblasts and enhanced deposition of collagen is seen in this disease, and it has been postulated that circulating cytokines are involved in the pathophysiology. Those, however, have not yet been characterized in detail. The involvement of the fingers in early disease may be mistaken as scleroderma, but the further development of the disease, the absence of antinuclear antibodies, and the lack of Raynaud’s phenomenon usually allow a clear diagnosis.

## Common Mechanisms

Sclerosing skin diseases represent a broad spectrum of clinically distinct diseases that, however, share common characteristics. Obviously, the specific nature of the ECM constituents produced by activated mesenchymal cells and the network formed by their interactions in these different disease entities determine the macromolecular structure and biomechanical properties of the microenvironment and thereby the characteristic clinical symptoms. Common to all is the activation of fibroblasts or their progenitor cells in the dermis (Figure 1). The initial triggers, however, are diverse and include external injuries such as infectious agents, drugs, toxins, or others. In most situations, the external triggers lead to activation of the innate and/or adaptive immune system. The resulting cytokine release is then responsible for the recruitment of mesenchymal progenitor cells and/or the activation of fibroblasts in the affected tissue. These cells have a diverse repertoire of receptors and signaling cascades to respond to specific inducers by modulating their biosynthetic pathways. Moreover, recent studies have demonstrated a high variability in fibroblast lineages, characterized by specific phenotypes. The data indicate that some are required for a fibrotic response, whereas others are probably involved in maintaining tissue integrity and/or connective tissue turnover (62). The type of trigger together with the specific response of fibroblasts in various differentiation states determines the quality of the accumulated ECM and the clinical phenotype.

The question to which extent a fibrotic reaction is reversible is still unclear. Probably the interaction of activated cells with the specific ECM in the microenvironment is an important factor together with the persistence of the release of fibrogenic cytokines. Another major determinant is the reversibility of ECM cross-links. Hence, the degree of reversibility and the role of metalloproteinases in tissue turnover are underestimated in many disease processes.

## Therapeutic Approaches

Certainly, the initial trigger should represent the best target to approach therapeutically. However, often these initial events are not sufficiently well characterized or they are difficult to counteract. Therefore, it is important to unravel the complex interplay between activation of cells of the innate and adaptive immune system, the release of cytokines, and growth factors and the response of the different fibroblast lineages. An in-depth understanding of these complex events might allow the development of therapies that target mechanisms common to several fibrosing skin diseases. Based on the growing understanding of these mechanisms, a number of agents have been developed that target specific pathways by modulating the Th1/Th2 immune response. Often these studies yielded a good response in animal models but have not shown sufficient efficacy in patient trials.

For all systemic therapeutic approaches aimed at influencing the fibrotic response, we have to consider that the target cells are embedded in an environment, in which locally bound growth factors, defined cell-ECM contacts, and tissue stiffness together determine the response to any therapeutic agent. The growing understanding of the cellular interactions and the significance of the ECM in this microenvironment allow the development of new concepts how to modulate these complex interplays and to target the activated cells.

## Author Contributions

All authors have contributed to drafting the manuscript, TK and BE wrote the final version, which was approved by all authors.

## Conflict of Interest Statement

The authors declare that the research was conducted in the absence of any commercial or financial relationships that could be construed as a potential conflict of interest.

## Abbreviations

αSMA, alpha smooth muscle actin; BMP, bone morphogenetic protein; CTGF, connective tissue growth factor; dcSSc, diffuse cutaneous systemic sclerosis; ECM, extracellular matrix; FGF, fibroblast growth factor; lcSSc, limited cutaneous systemic sclerosis; PDGF, platelet-derived growth factor; TGFβ, transforming growth factor beta.
